# Radiomics model based on vertebral calcium-suppressed CT images for predicting chemotherapy-induced myelosuppression in nasopharyngeal carcinoma

**DOI:** 10.3389/fonc.2025.1574250

**Published:** 2025-09-03

**Authors:** Yanhui Yang, Jing Hou, Yi Zhang, Yan Sun, Yi Fu, Qiang Lu, Tao Luo, Zhijie Huang, Xiaoping Yu

**Affiliations:** ^1^ Department of Diagnostic Radiology, the Affiliated Cancer Hospital of Xiangya School of Medicine, Central South University/Hunan Cancer Hospital, Changsha, Hunan, China; ^2^ Department of Diagnostic Radiology, Graduate Collaborative Training Base of Hunan Cancer Hospital, Hengyang Medical School, University of South China, Hengyang, Hunan, China; ^3^ The First Affiliated Hospital, Department of Radiology, Hengyang Medical School, University of South China, Hengyang, China; ^4^ Medical Department, the Affiliated Cancer Hospital of Xiangya School of Medicine, Central South University/Hunan Cancer Hospital, Changsha, Hunan, China; ^5^ Clinical and Technical Support, Philips Healthcare, Guangzhou, China

**Keywords:** dual-layer computed tomography (DLCT), myelosuppression, nasopharyngeal carcinoma, calcium-suppressed (CaSupp) imaging, radiomics

## Abstract

**Objective:**

To develop and validate a radiomics model based on vertebral calcium-suppressed (CaSupp) images derived from dual-layer computed tomography (DLCT) for predicting chemotherapy-induced myelosuppression in patients with locally advanced nasopharyngeal carcinoma (LANPC).

**Methods:**

This retrospective study included 150 LANPC patients treated with induction chemotherapy (IC). Radiomics features were extracted from lumbar vertebral CaSupp obtained from baseline DLCT scans. Models were developed to predict myelosuppression after the first chemotherapy cycle (IC - 1) and entire chemotherapy cycles (IC-n). The clinics, radiomics, and combined models were conducted via multivariate logistic regression. Models performance was evaluated by the area under the receiver operating characteristic curve (AUC). Clinical utility was analyzed with decision curve analysis.

**Results:**

For predict myelosuppression after IC - 1, the clinics, radiomics, and combined models had AUC values of 0.716, 0.825 and 0.859 in the train cohort, respectively; and AUC of 0.687, 0.752 and 0.790 in the test cohort, respectively. And for IC-n, the clinics, radiomics, and combined models exhibited AUC values of 0.771, 0.824, and 0.889 in the train cohort, respectively; and AUC of 0.652, 0.740 and 0.806 in the test cohort, respectively. For predicting myelosuppression after both IC - 1 and IC-n,the combined models demonstrated significantly higher AUC values than the clinics models for both IC - 1 and IC-n (all P<0.05).

**Conclusions:**

Radiomics model based on vertebral CaSupp images from DLCT could predict chemotherapy-induced myelosuppression in LANPC patients. This study highlights the potential of DLCT technology to provide quantitative bone marrow assessments and aid in personalized treatment planning. External validation and comparison with other imaging modalities are warranted in the future.

## Introduction

Nasopharyngeal carcinoma (NPC) is a prevalent head and neck malignancy in East and Southeast Asia (1), with over 70% of new diagnoses in the locally advanced stage (III-IVa) (2). For patients with locally advanced nasopharyngeal carcinoma (LANPC), the primary treatment approach is concurrent chemoradiotherapy following induction chemotherapy (IC) (3). However, while platinum-based drugs effectively eliminate tumor cells, they also inhibit or destroy simultaneously normal proliferating cells, such as hematopoietic stem and progenitor cells in the bone marrow (4). Chemotherapy-induced myelosuppression is one of the most common hematological and dose-limiting toxic reactions (5). A recent study showed that all patients with LANPC experience different degrees of myelosuppression during IC (6). Myelosuppression primarily manifests leukopenia, neutropenia, anemia, and thrombocytopenia, leading to infections, fatigue, and bleeding, potentially delaying treatment schedules, reducing the therapeutic dosage, and affecting treatment efficacy (7–9). Therefore, predicting myelosuppression occurrence before patients undergo IC could alleviate patient burden and improve treatment outcomes. The response to hematologic toxicity induced by chemotherapy varies among patients, thus necessitating the establishment of predictive models for myelosuppression risk. Up to now, research on predicting myelosuppression induced by chemotherapy mainly focused on clinical factors, such as age, laboratory results, etc., and demonstrated good predictive performance (10–13). However, the investigation involved in predicting myelosuppression after chemotherapy for NPC is scarce, whether based on clinical or imaging data.

Radiomics aims to convert medical images into high-dimensional, quantitative imaging features, thereby improving disease management in a cost-effective and non-invasive manner (14). Radiomics models based on pre-radiotherapy CT images of NPC have shown promising performance in predicting lymphopenia (15). For example, Ren et al. (16) demonstrated that radiomics models based on pre-radiotherapy CT images in cervical cancer patients have predictive value for hematological toxic reactions.

Dual-layer detector CT (DLCT) is an emerging imaging technology that acquires two sets of image data with high and low energy in a single image acquisition, enabling the virtual removal of specific materials (e.g. calcium) (17). Based on calcium suppression algorithms, calcium-suppressed (CaSupp) imaging can identify and virtually remove calcium content (18, 19), allowing for the estimation of fat and soft tissue components in the bone marrow (19, 20). CaSupp technology eliminates the influence of calcium, making it potentially valuable for describing vertebral bone marrow (17–19). Reportedly, texture features extracted from CaSupp images obtained before and after treatment are correlated with specific hematological parameters in patients with multiple myeloma (21, 22), indicating the potential value of radiomics models from DLCT-based CaSupp images in predicting hematological toxicity of chemotherapy.

Therefore, this study aims to develop a radiomics model utilizing DLCT-derived CaSupp images to predict the occurrence of myelosuppression after chemotherapy in LANPC, to help guide clinical practice in predicting the hematological response to chemotherapy.

## Materials and methods

### Patients

**Figure 1:**
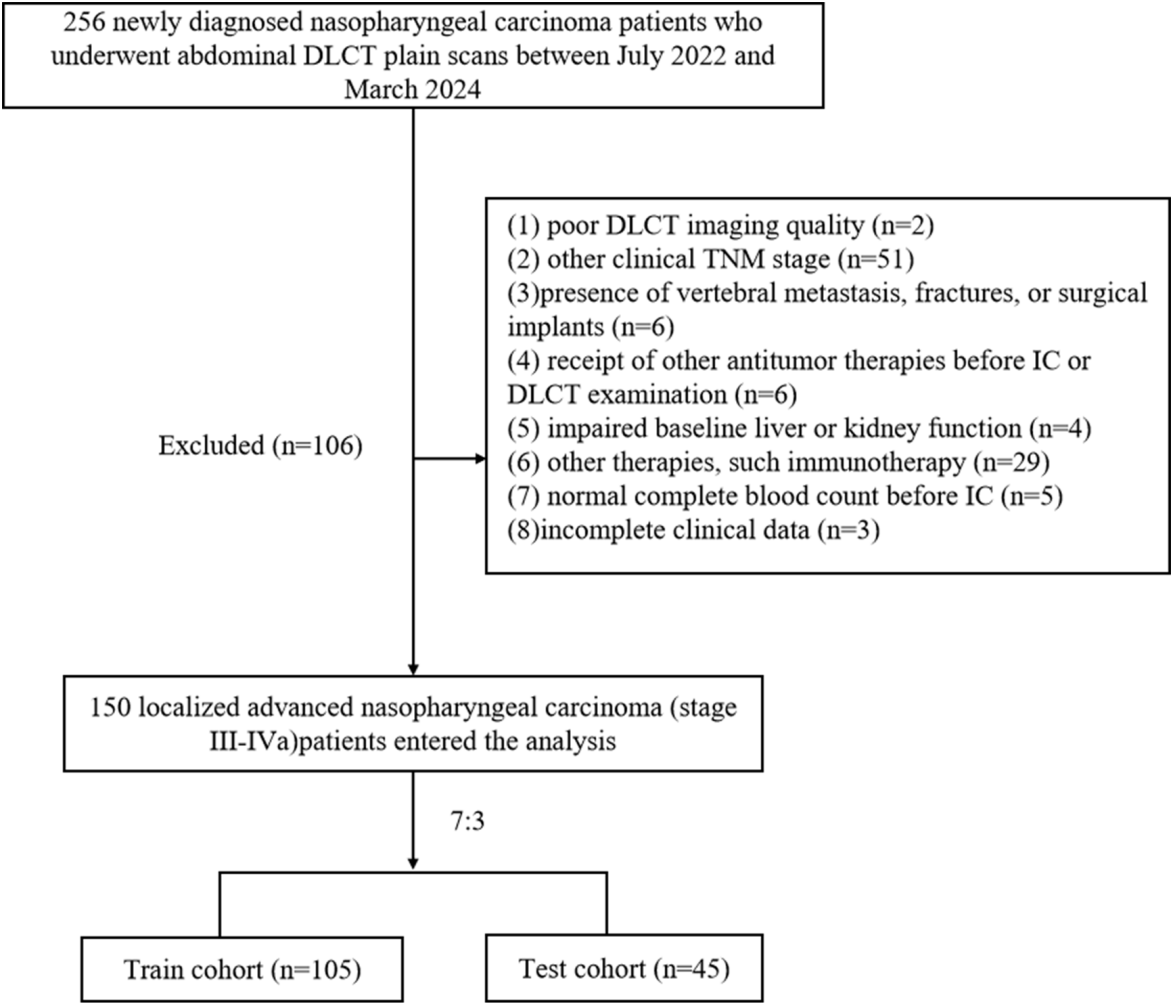
Flowchart for patients’ selection.

The ethics committee of our hospital approved this study. This was a retrospective study based on routine CT examination and clinical data analysis, so patients’ individual written informed consent was not required. We retrospectively analyzed NPC patients who underwent non-enhanced abdominal DLCT examination in our hospital from July 2022 to March 2024. Inclusion criteria were as follows: (1) age over 18 years; (2) pathologically confirmed LANPC; (3) CT scanning range included all lumbar vertebrae; (4) received at least one cycle of IC, regardless of subsequent concurrent chemoradiotherapy. Exclusion criteria included: (1) poor DLCT imaging quality (n=2); (2) other clinical TNM stage (n=51);(3)presence of vertebral metastasis, fractures, or surgical implants (n=6); (4) receipt of other antitumor therapies before IC or DLCT examination (n=6); (5) impaired baseline liver or kidney function (n=4),defined as alanine aminotransferase or aspartate aminotransferase levels > 40 U/L, and serum creatinine > 106 μmol/L in males or > 97 μmol/L in females, according to our hospital’s laboratory reference ranges; (6) other therapies, such immunotherapy (n=29); (7) normal complete blood count before IC (n=5); or (8) incomplete clinical data (n=3). NPC staging for all patients followed the 8th edition of the American Joint Committee on Cancer TNM staging system. A total of 256 NPC patients were initially screened, and 150 cases were ultimately included (**Figure 1**).

### Clinical data

We collected data before IC from the hospital medical record information system, including (1) baseline clinical information: gender, age, smoking history, drinking history, hypertension history, diabetes history, body mass index (BMI), tumor pathology type, clinical TNM stage, chemotherapy regimen, etc.; and (2) laboratory indices in baseline and IC periods: liver and kidney function, complete blood count, neutrophil-to-lymphocyte ratio (NLR), platelet-to-lymphocyte ratio (PLR), lymphocyte-to-monocyte ratio (LMR), and prognostic nutritional index (PNI), where PNI is defined as serum albumin (g/L) + 5× total lymphocyte count (10^9/L). In this study, the assessment of myelosuppression was based on the blood routine data collected from the medical record information system after IC. According to the Common Terminology Criteria for Adverse Events (CTCAE) version 5.0 from the National Cancer Institute, the occurrence of myelosuppression is defined as the observation of any of the following indicators in peripheral blood samples during IC: Leukocyte count < 4×10^9/L, Neutrophil count < 2×10^9/L, Platelet count < 100×10^9/L, or Hemoglobin count < 110g/L. Our study endpoints were the incidence of myelosuppression during the first cycle (IC - 1) and the entire cycle of induction chemotherapy (IC-n). Here, IC - 1 and IC-n referred to the occurrence of myelosuppression in the first and any cycle of the IC treatment, respectively.

All treatment regimens for patients adhered to the first-line therapeutic recommendations outlined in the National Comprehensive Cancer Network (NCCN) Clinical Practice Guidelines in Oncology for head and neck cancers(3), with the GP (Gemcitabine and Cisplatin) or TPF (Docetaxel, Cisplatin and 5-Fluorouracil) regimens being the preferred IC strategies for LANPC. Concurrently, the current guidelines of the Chinese Society of Clinical Oncology (CSCO) for NPC recommend the GP, TPF, and TP (Docetaxel and Cisplatin) regimen as the initial IC options (23). The selection of the IC regimen was contingent upon the patient’s clinical profile, the therapeutic directives from both the NCCN and CSCO, as well as the patient’s choice. Following the treatment protocols of our institution, a total of three distinct platinum-based chemotherapy regimens were included, encompassing the GP (n=56), TPF (n=41),and TP (n= 53) regimen for 1 to 4 chemotherapy cycles.

### Image acquisition, segmentation and radiomics feature extraction

Patients underwent abdominal DLCT scans (IQon Spectral CT; Philips Healthcare), covering all lumbar vertebrae. Scanning parameters followed the factory default scan protocol: tube voltage, 120 kV; adaptive tube current; pitch, 0.5; gantry rotation time, 0.5s; and section collimation 64×0.625 mm. After completing the scanning process, the acquired data was projected for spatial-spectral reconstruction (Spectral level 4), with a thickness and an image spacing of 0.992mm. Calcium suppression index (CaSupp-I) of 25% was automatically generated from the Spectral Base Image data. The second and the fourth lumbar vertebrae were selected as the target vertebrae, which referred to a previous study (25). Subsequently, in the reconstructed image with CaSupp-I 25%, the region of interest (ROI) was delineated manually in the cancellous area at the axial plane of the vertebral body, centered between midvertebral and the superior endplate, using 3D-slicer software (version 5.6, https://www.slicer.org/).All procedures were conducted by a radiologist with five years of clinical experience in diagnostic imaging. Care was taken to avoid including cortical bone.

Radiomic**s** features were extracted from ROIs of the two vertebrae in CaSupp-I 25% image, which had an original axial imaging matrix of 512×512. Preprocessing was performed to ensure image comparability, including: (1) resampling images to a voxel size of 1×1×1 mm^3^; (2) discretizing grayscale values with a bin width of 25 gray levels (26); and (3) normalizing grayscale values across all images to a uniform range of [0,1] using PyRadiomics (normalize = True, normalizeScale = 50), applying a Z‐score transform f(x)=50 (x−μ)/σ to all voxels (27).

### Radiomics feature selection and radiomics construction

**Figure 2:**
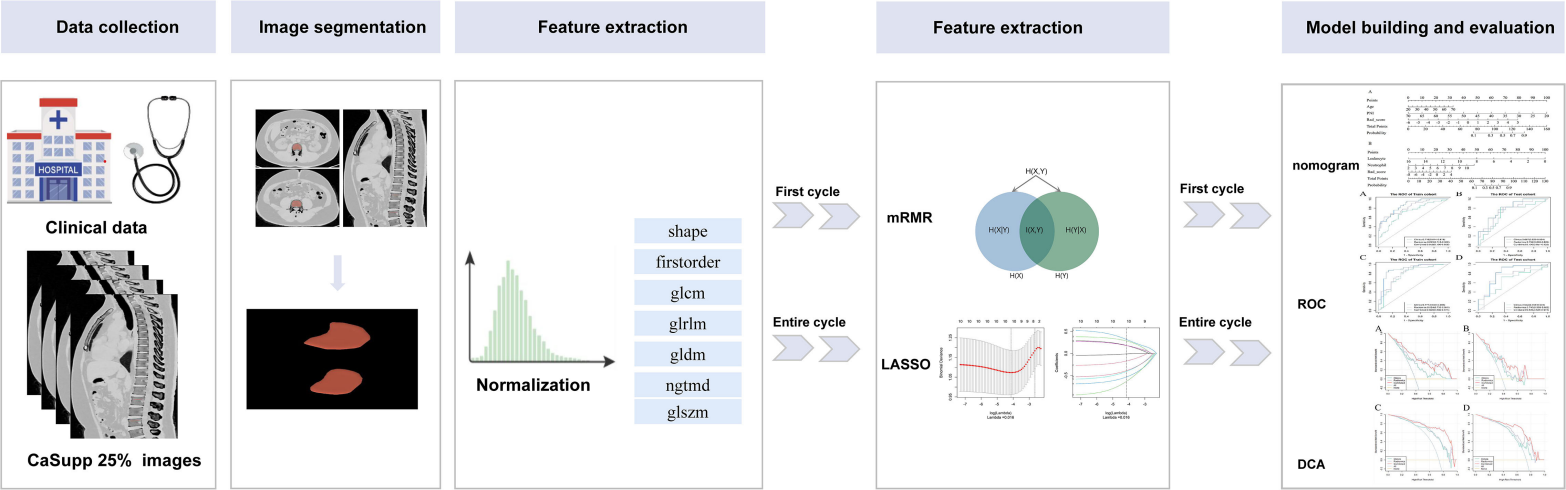
Flowchart of the radiomics model.

The 150 subjects were randomly divided in a 7:3 ratio into the train (N = 105) and test cohorts (N = 45). The following methods were conducted in the train cohort to identify the most stable and predictive features for constructing the final radiomics model: maximum relevance minimum redundancy (mRMR) (28)algorithm and least absolute shrinkage and selection operator (LASSO) regression (29). Specifically, all extracted features were first imported into the mRMR algorithm for feature selection according to different endpoints, with redundant and irrelevant features eliminated. Subsequently, the LASSO analysis was applied to the features selected previously to determine the optimal features for establishing the radiomics model. Considering the potential interrelationships between radiomics features rather than their independence, the selected features were combined by adding the product of each feature and its corresponding regression coefficient to calculate the radiomics score (Rad_score). The workflow for image segmentation, feature selection, model construction, and model evaluation are illustrated in **Figure 2**.

### Clinical and combined models construction and performance evaluation

In the train cohort, univariate and multivariate regression analyses were performed to screen predictive clinical variables. Variables with P < 0.05 in the univariate analysis were included in a backward multivariate regression, and subsequently, these selected variables were utilized to construct the clinics models. Meanwhile, these clinical variables, along with the Rad_score, were incorporated to construct the combined models for predicting myelosuppression. Nomograms were generated to visualize the combined models. The Hosmer-Lemeshow test was used to assess the goodness-of-fit of the models and calibration curves were employed to visualize model fit. Additionally, decision curve analysis (DCA) was used to evaluate the clinical utility of the models.

### Statistical analysis

All statistical analyses were conducted using R software (version 4.0.2). Feature selection through mRMR and LASSO was performed with the “mRMRe” and “glmnet” packages, respectively. Normality of continuous variables was assessed using the Shapiro–Wilk test. Based on the results of normality tests, continuous data were compared between groups using either the t-test or the Wilcoxon test. For categorical variables, group differences were analyzed using the chi-square test, or Fisher’s exact test when applicable. The diagnostic performance of different models was assessed by calculating the area under the receiver operating characteristic curve (AUC). To compare the predictive accuracy of our radiomics models, pairwise AUC differences were assessed by DeLong’s test, and P-values were adjusted using the Bonferroni correction to maintain an overall family-wise error rate of 0.05 (i.e., per-comparison α = 0.05/number_of_tests). A two-tailed P-value <0.05 was considered statistically significant.

## Results

### Clinical characteristics

A total of 150 participants (101 males and 49 females), aged between 21 and 70 years, were enrolled in the study. The occurrence rates of myelosuppression in IC - 1 and IC-n were 38.67% (58/150) and 73.33% (110/150), respectively. Compared with the non-myelosuppression group, the myelosuppression group had significantly lower baseline Leukocyte count, Neutrophil count, Lymphocyte count, and PNI (all P<0.05, **Table 1**). Additionally, for IC - 1, patients experiencing myelosuppression were found to be older than those without myelosuppression; for the IC-n, the myelosuppression group showed a higher PLR, a lower LMR, and a more advanced clinical TNM stage than the non-myelosuppression group (all P<0.05, **Table 1**).

**Table 1:** Baseline clinical characteristics for patients with or without myelosuppression in the first and the entire induction chemotherapy cycle.

Variables	Total (n = 150)	First cycle (IC - 1)			Entire cycle (IC-n)		
		Non-myelosuppression (n = 92)	Myelosuppression (n = 58)	P	Non-myelosuppression (n = 40)	Myelosuppression (n = 110)	P
Age (Year)	49.24 ± 9.39	47.83 ± 9.96	51.48 ± 7.98	0.020	47.55 ± 11.74	49.85 ± 8.35	0.259
Gender (male, %)	101 (67.33)	63 (68.48)	38 (65.52)	0.706	27 (67.50)	74 (67.27)	0.979
BMI (kg/m²),	23.73 (21.67, 25.76)	23.73 (21.51, 26.62)	23.67 (22.05, 24.90)	0.359	23.72 (22.55, 26.75)	23.77 (21.37, 25.38)	0.258
Creatinine (μmol/L)	68.04 ± 12.79	68.38 ± 13.57	67.51 ± 11.54	0.688	68.15 (58.00, 84.60)	68.50 (58.05, 74.90)	0.129
Serum Albumin(g/L)	42.90 (40.80, 44.77)	42.95 (41.18, 45.15)	42.85 (40.30, 44.58)	0.234	42.75 (41.60, 44.92)	43.05 (40.73, 44.70)	0.429
PT(s)	11.90 (11.40, 12.38)	11.90 (11.28, 12.30)	12.00 (11.50, 12.47)	0.228	11.70 (11.17, 12.40)	11.95 (11.50, 12.30)	0.416
APTT(s)	31.55 (27.57, 35.10)	31.05 (27.50, 34.78)	32.30 (28.68, 35.27)	0.504	31.20 (27.45, 34.80)	32.20 (28.50, 35.18)	0.652
TT(s)	16.75 (16.10, 17.40)	16.70 (15.90, 17.22)	16.90 (16.20, 17.50)	0.111	16.80 (16.23, 17.50)	16.70 (16.10, 17.38)	0.843
Hemoglobin count (g/L)	144.73 ± 15.86	146.01 ± 16.53	142.69 ± 14.65	0.213	147.80 ± 15.82	143.61 ± 15.80	0.153
Leukocyte count (g/L)	6.50 (5.57, 7.92)	6.87 (5.81, 8.24)	5.88 (5.24, 7.45)	0.002	7.63 (6.42, 9.24)	6.25 (5.41, 7.45)	<.001
Neutrophil count (×10^9^/L)	4.06 (3.26, 5.04)	4.21 (3.38, 5.25)	3.76 (3.00, 4.68)	0.032	4.49 (3.64, 5.47)	3.92 (3.11, 4.78)	0.010
Monocyte count (×10^9^/L)	0.49 (0.37, 0.59)	0.52 (0.39, 0.61)	0.48 (0.34, 0.55)	0.090	0.55 (0.41, 0.61)	0.48 (0.35, 0.57)	0.061
Platelet count (×10^9^/L)	241.00 (210.25, 282.75)	247.00 (213.75, 287.75)	233.00 (202.00, 270.25)	0.153	246.50 (213.75, 293.25)	236.00 (207.00, 271.00)	0.178
Lymphocyte count (×10^9^/L)	1.94 (1.47, 2.43)	2.03 (1.53, 2.55)	1.75 (1.34, 2.16)	0.018	2.34 (1.91, 2.84)	1.73 (1.41, 2.18)	<.001
NLR	2.14 (1.71, 2.84)	2.06 (1.68, 2.85)	2.19 (1.77, 2.83)	0.569	1.84 (1.56, 2.43)	2.23 (1.75, 2.87)	0.094
PLR	125.33 (100.23, 170.04)	123.16 (97.42, 161.11)	134.45 (105.27, 180.00)	0.176	116.89 (84.25, 145.13)	129.92 (107.01, 180.00)	0.015
SII	520.70 (361.50, 743.45)	489.06 (371.38, 737.94)	543.62 (349.25, 743.45)	0.912	477.21 (372.28, 692.66)	546.14 (360.11, 758.06)	0.642
PNI	52.50 (50.10, 55.56)	53.10 (50.90, 56.35)	51.20 (49.41, 53.60)	0.004	54.83 (52.12, 56.50)	51.42 (49.49, 54.56)	<.001
LMR	4.14 (3.22, 4.87)	4.33 (3.28, 4.90)	3.77 (3.05, 4.76)	0.218	4.65 (3.81, 5.23)	3.73 (3.12, 4.76)	0.010
Smoking (+, %)	71 (47.33)	45 (48.91)	26 (44.83)	0.626	20 (50.00)	51 (46.36)	0.693
Drinking (+, %)	52 (34.67)	33 (35.87)	19 (32.76)	0.697	13 (32.50)	39 (35.45)	0.737
Hypertension (+, %)	24 (16.00)	19 (20.65)	5 (8.62)	0.050	7 (17.50)	17 (15.45)	0.763
Diabetes (+, %)	4 (2.67)	3 (3.26)	1 (1.72)	0.961	2 (5.00)	2 (1.82)	0.619
EB concentration(copies/ml)				0.588			0.498
0-999	115 (76.67)	68 (73.91)	47 (81.03)		30 (75.00)	85 (77.27)	
1000-9999	31 (20.67)	21 (22.83)	10 (17.24)		10 (25.00)	21 (19.09)	
≥10000	4 (2.67)	3 (3.26)	1 (1.72)		0 (0.00)	4 (3.64)	
Clinical T stage				0.117			0.333
1	10 (6.67)	8 (8.70)	2 (3.45)		5 (12.50)	5 (4.55)	
2	37 (24.67)	18 (19.57)	19 (32.76)		8 (20.00)	29 (26.36)	
3	64 (42.67)	38 (41.30)	26 (44.83)		16 (40.00)	48 (43.64)	
4	39 (26.00)	28 (30.43)	11 (18.97)		11 (27.50)	28 (25.45)	
Clinical N stage				0.901			0.256
0	3 (2.00)	2 (2.17)	1 (1.72)		1 (2.50)	2 (1.82)	
1	28 (18.67)	18 (19.57)	10 (17.24)		8 (20.00)	20 (18.18)	
2	59 (39.33)	34 (36.96)	25 (43.10)		11 (27.50)	48 (43.64)	
3	60 (40.00)	38 (41.30)	22 (37.93)		20 (50.00)	40 (36.36)	
Clinical TNM stage				0.113			0.023
III	68 (45.33)	37 (40.22)	31 (53.45)		12 (30.00)	56 (50.91)	
IVa	82 (54.67)	55 (59.78)	27 (46.55)		28 (70.00)	54 (49.09)	
Pathological type				1			0.388
WHO II	10 (6.67)	6 (6.52)	4 (6.90)		1 (2.50)	9 (8.18)	
WHO III	140 (93.33)	86 (93.48)	54 (93.10)		39 (97.50)	101 (91.82)	

BMI, Body Mass Index.PT, Prothrombin Time. APTT, Activated Partial Thromboplastin Time. TT, Thrombin Time. NLR, Neutrophil-to-lymphocyte ratio. PLR, Platelet-to-lymphocyte ratio. SII, Systemic immune-inflammation index. LMR, Lymphocyte-to-monocyte ratio. PNI= Serum albumin value (g/L) + 5× Total number of peripheral blood lymphocytes count (×10^9^/L). WHO, World Health Organization. Measurement data were expressed as mean ± standard deviation or median (interquartile range), and categorical variables are expressed by quantity (percentage). T-test or rank sum test for count data, and chi square test or Fisher exam for categorical data.

### Radiomics model construction

A total of 1130 features were extracted from the vertebral ROIs, categorized into three types: shape-based features, first-order statistical features, and texture features which include gray-level co-occurrence matrix (GLCM), gray-level run length matrix (GLRLM), gray-level size zone matrix (GLSZM), gray-level dependence matrix (GLDM) and neighborhood gray-tone difference matrix (NGTDM). Based on the IC - 1 and IC-n endpoints, 10 features most relevant to the outcomes were ultimately selected by the mRMR and LASSO algorithms (**Supplementary Table S1**). These features were used to calculate the Rad_score values and develop the radiomics and combined models.

### Radiomics feature selection

The myelosuppression group had significantly lower Rad_score values than the non-myelosuppression group (-1.22 vs 0.59 for IC - 1, P<0.001; and 0.11 vs 1.85 for IC-n, P<0.001). Among the 10 features radiomics selected to calculate the Rad_score values, the most strongly associated with myelosuppression for IC - 1 were wavelet.LLH_glszm_SizeZoneNonUniformityNormalized (Feature 1) and wavelet.HHL_firstorder_Mean (Feature 2). For IC-n, the most relevant features were original_glcm_InverseVariance (Feature 3) and wavelet.LHH_glszm_SizeZoneNonUniformityNormalized (Feature 4). The median values of these features in the myelosuppression and non-myelosuppression groups were as follows: 0.228 vs. 0.216 (Feature 1; P = 0.003), -0.163 vs. -0.121 (Feature 2; P = 0.007), 0.479 vs. 0.489 (Feature 3; P = 0.039), and 0.293 vs. 0.285 (Feature 4; P = 0.031).

### Clinics and combined models construction

Multivariate logistic regression analyses indicated that age and PNI were independent predictors of myelosuppression for IC - 1, while Leukocyte and Neutrophil count emerged as independent predictors for IC-n (**Table 2**). These clinical predictors were used to develop the clinics models.

**Table 2:** Clinical risk factors for myelosuppression in the first and entire induction chemotherapy cycle.

Variable	Univariate logistic regression		Multivariate logistic regression	
	P	Odds ratio (95%CI)	P	Odds ratio (95%CI)
First cycle (IC - 1)				
Age	0.022	1.05 (1.01 ~ 1.09)	0.039	1.04 (1.01 ~ 1.08)
Leukocyte count	0.023	0.80 (0.66 ~ 0.97)	–	–
Lymphocyte count	0.022	0.54 (0.32 ~ 0.92)	–	–
PNI	0.006	0.90 (0.83 ~ 0.97)	0.01	0.91 (0.84 ~ 0.98)
Entire cycle (IC-n)				
Leukocyte count	<.001	0.71 (0.58 ~ 0.87)	<.001	0.42 (0.25 ~ 0.70)
Neutrophil count	0.022	0.76 (0.60 ~ 0.96)	0.024	2.07 (1.10 ~ 3.89)
Lymphocyte count	<.001	0.33 (0.19 ~ 0.59)	–	–
PLR	0.026	1.01 (1.01 ~ 1.02)	–	–
PNI	<.001	0.86 (0.78 ~ 0.94)	–	–
Clinical TNM stag				
III		1.00 (Reference)	–	–
IVa	0.025	0.41 (0.19 ~ 0.89)	–	–

Only clinical variables with P<0.05 in univariate analysis were shown. PNI= Serum albumin value (g/L) + 5× Total number of peripheral blood lymphocytes count (×10^9^/L). PLR, Platelet-to-lymphocyte ratio.

**Figure 3:**
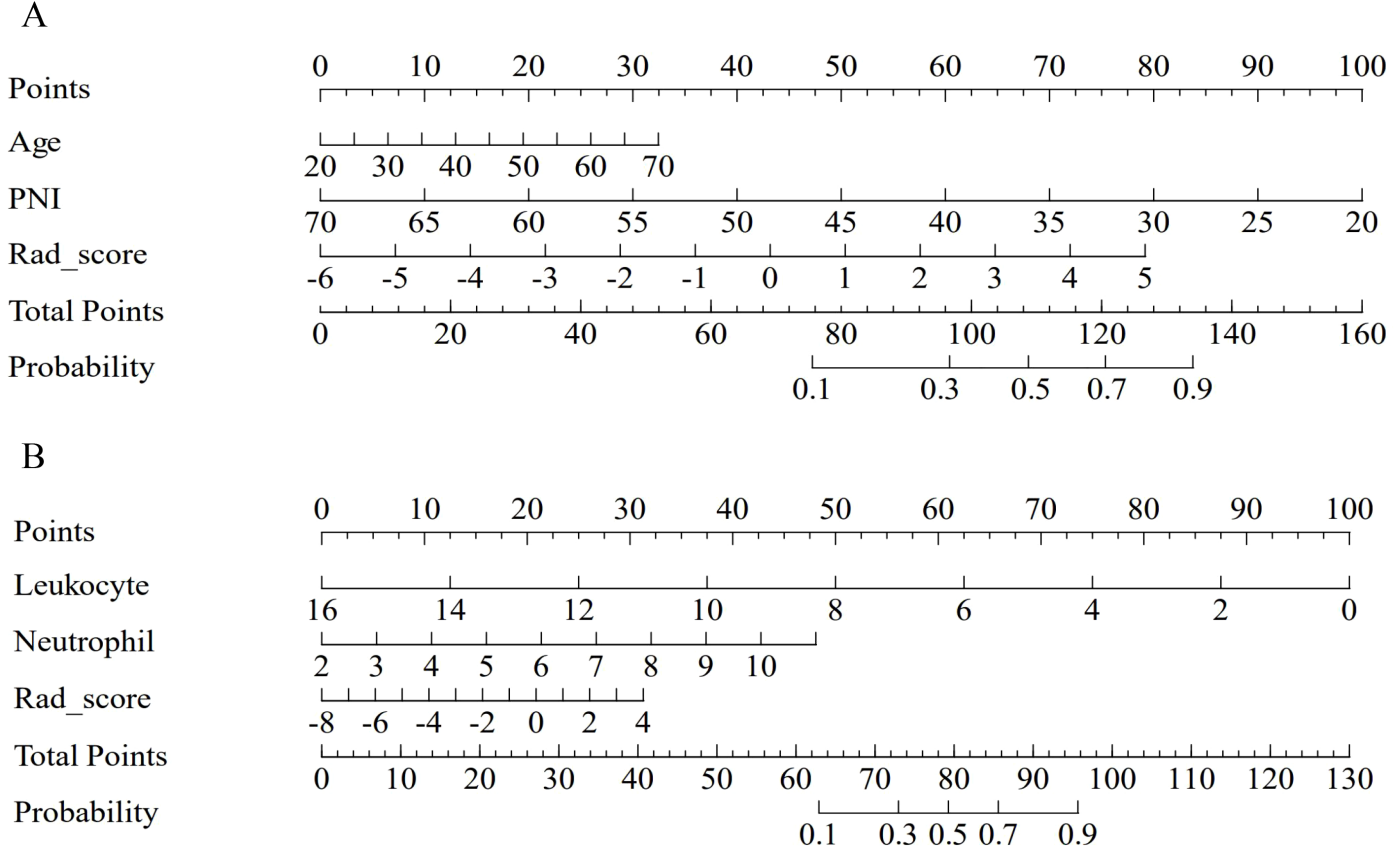
The nomogram of the combined model for predicting myelosuppression. The nomogram of the combined model integrates clinical variables and Rad_score for predicting myelosuppression in the first **(A)** and the entire **(B)** induction chemotherapy cycle in the train cohort.


The nomograms integrating radiomics features and clinical variables are shown in **Figure 3**. As illustrated, the nomogram visualizes the combined model’s predicted risk using a point scale. The predictive formulas for each endpoint are as follows:

$$Y_{IC-1}=2.73252+0.03381*Age\left(Years\right)-0.10418*PNI+0.37493*Rad\_score_{IC-1}$$

$$Y_{IC-n}=4.1441-0.8383*Leukocyte\left(10^{9}/L\right)+0.7166*Neutrophil\left(10^{9}/L\right)+0.3498*Rad\_score_{IC-n}$$

The specific calculation formulas for Rad_score corresponding to IC - 1 and IC-n are provided in **Supplementary Table S1**.

**Figure 4:**
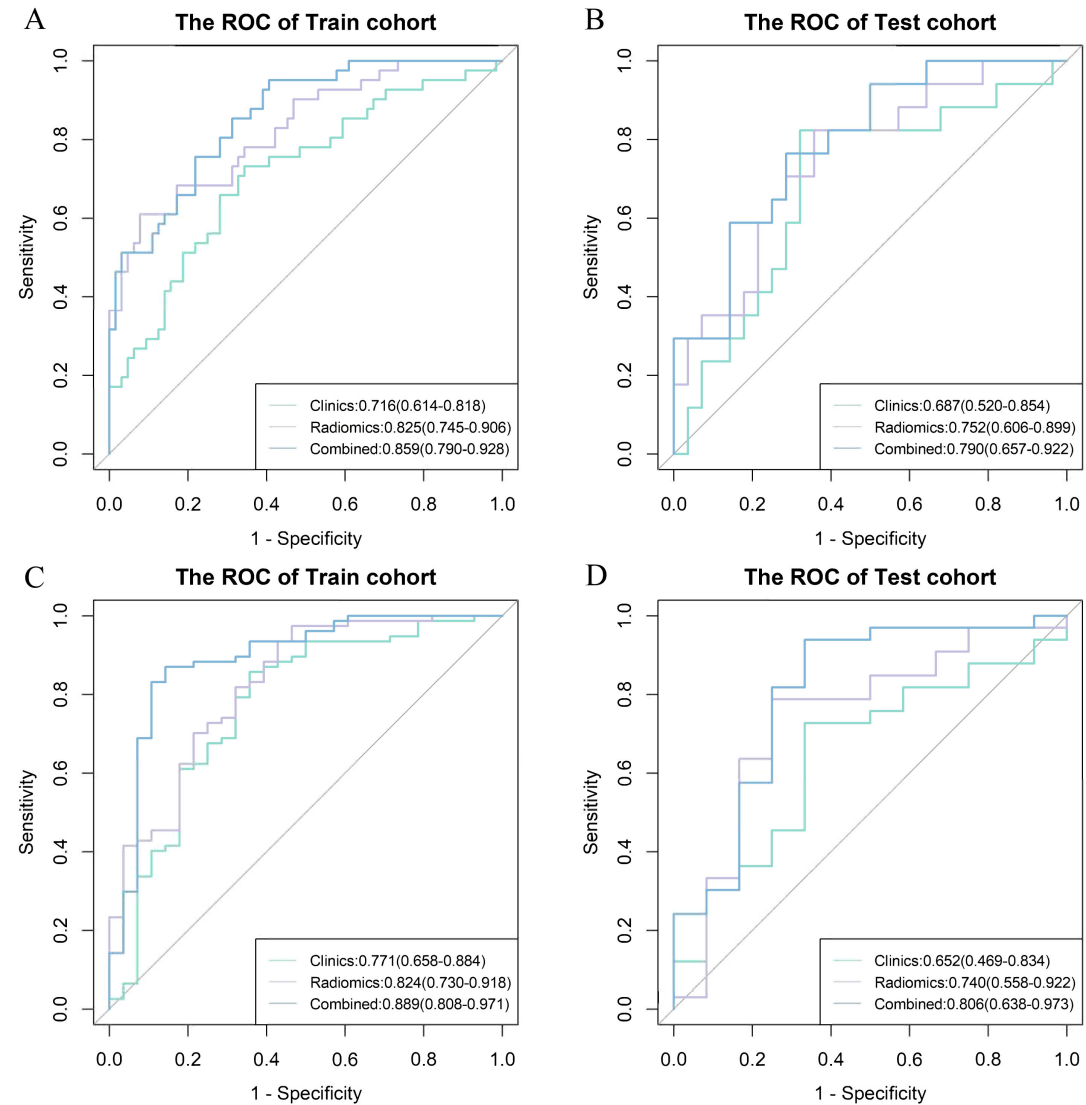
Receiver operating characteristic (ROC) curve among different models for the prediction of myelosuppression. ROC curve among clinics, radiomics and combined model for the prediction of myelosuppression in the train **(A)** and test **(B)** cohort in the first induction chemotherapy cycle, and the train **(C)** and test **(D)** cohort in the entire induction chemotherapy cycle.

The combined model exhibited good predictive accuracy, achieving AUC values for IC - 1 of 0.859 (95% CI: 0.790 - 0.928) in the train cohort and 0.79 (95% CI: 0.657 - 0.922) in the test cohort. Similarly, for IC-n, the AUC values were 0.889 (95% CI: 0.808 - 0.971) and 0.806 (95% CI: 0.638 - 0.973) in the train and test cohorts, respectively (**Table 3**, **Figure 4**).

**Table 3:** Diagnostic performances of the models for predicting myelosuppression in the first and entire induction chemotherapy cycle.

Models	Train cohort				Test cohort			
	AUC (95%CI)	Sensitivity (95%CI)	Specificity (95%CI)	Accuracy (95%CI)	AUC (95%CI)	Sensitivity (95%CI)	Specificity (95%CI)	Accuracy (95%CI)
First cycle (IC - 1)								
Clinics model	0.716 (0.614 - 0.818)	0.651 (0.533 - 0.769)	0.744 (0.7607 - 0.881)	0.686 (0.587 - 0.775)	0.687 (0.520 - 0.845)	0.643 (0.465 - 0.820)	0.792 (0.642 - 0.842)	0.694 (0.581 - 0.811)
Radiomics model	0.825 (0.745 - 0.906)	0.921 (0.856 - 0.987)	0.559 (0.435 - 0.744)	0.800 (0.797 - 0.803)	0.752 (0.606 - 0.899)	0.679 (0.506 - 0.852)	0.804 (0.642 - 0.898)	0.711 (0.557 - 0.836)
Combined model	0.859 (0.790 - 0.928)	0.603 (0.482 - 0.724)	0.949 (0.8779 - 1.000)	0.810 (0.807 - 0.812)	0.790 (0.657 - 0.922)	0.714 (0.547 - 0.882)	0.765 (0.563 - 0.966)	0.733 (0.581 - 0.854)
Entire cycle (IC-n)								
Clinics model	0.771 (0.658 - 0.884)	0.643 (0.465 - 0.820)	0.857 (0.779 - 0.935)	0.800 (0.711 - 0.820)	0.652 (0.469 - 0.834)	0.667 (0.400 - 0.933)	0.727 (0.575 - 0.879)	0.710 (0.702 - 0.720)
Radiomics model	0.824 (0.730 - 0.918)	0.536 (0.351 - 0.720)	0.974 (0.938 - 1.000)	0.857 (0.775 - 0.918)	0.740 (0.558 - 0.922)	0.750 (0.505 - 0.995)	0.788 (0.648 - 0.927)	0.778 (0.770 - 0.785)
Combined model	0.889 (0.808 - 0.971)	0.857 (0.728 - 0.987)	0.870 (0.795 - 0.945)	0.867 (0.786 - 0.925)	0.806 (0.638 - 0.973)	0.706 (0.485 - 0.913)	0.939 (0.858 - 1.000)	0.867 (0.732 - 0.949)

AUC, Area under ROC curve; 95% CI, 95% confidence interval.

### Models evaluation and comparison

For IC - 1, the clinics model, radiomics model, and combined model had AUC values of 0.716,0.825 and 0.859 in the train cohort, respectively; and AUC of 0.687, 0.752 and 0.790 in the test cohort, respectively. For IC-n, the clinics model, radiomics model, and combined model exhibited AUC values of 0.771, 0.824, and 0.889 in the train cohort, respectively; and AUC of 0.652, 0.740 and 0.806 in the test cohort, respectively (**Table 3**, **Figure 4**).

The combined models demonstrated significantly higher AUC values than the clinics models for both IC - 1 and IC-n (all P<0.05). The combined models showed significantly higher AUC values than the radiomics models for IC-n (P<0.05), but not for IC - 1. The differences in the AUC values between the radiomics and clinics models tended to reach statistical significance for IC - 1 (P = 0.086 and 0.093), but not for IC-n (**Table 3**, **Supplementary Table S2**). However, for both IC - 1 and IC-n, the radiomics models demonstrated higher accuracy than the clinics models.

**Figure 5:**
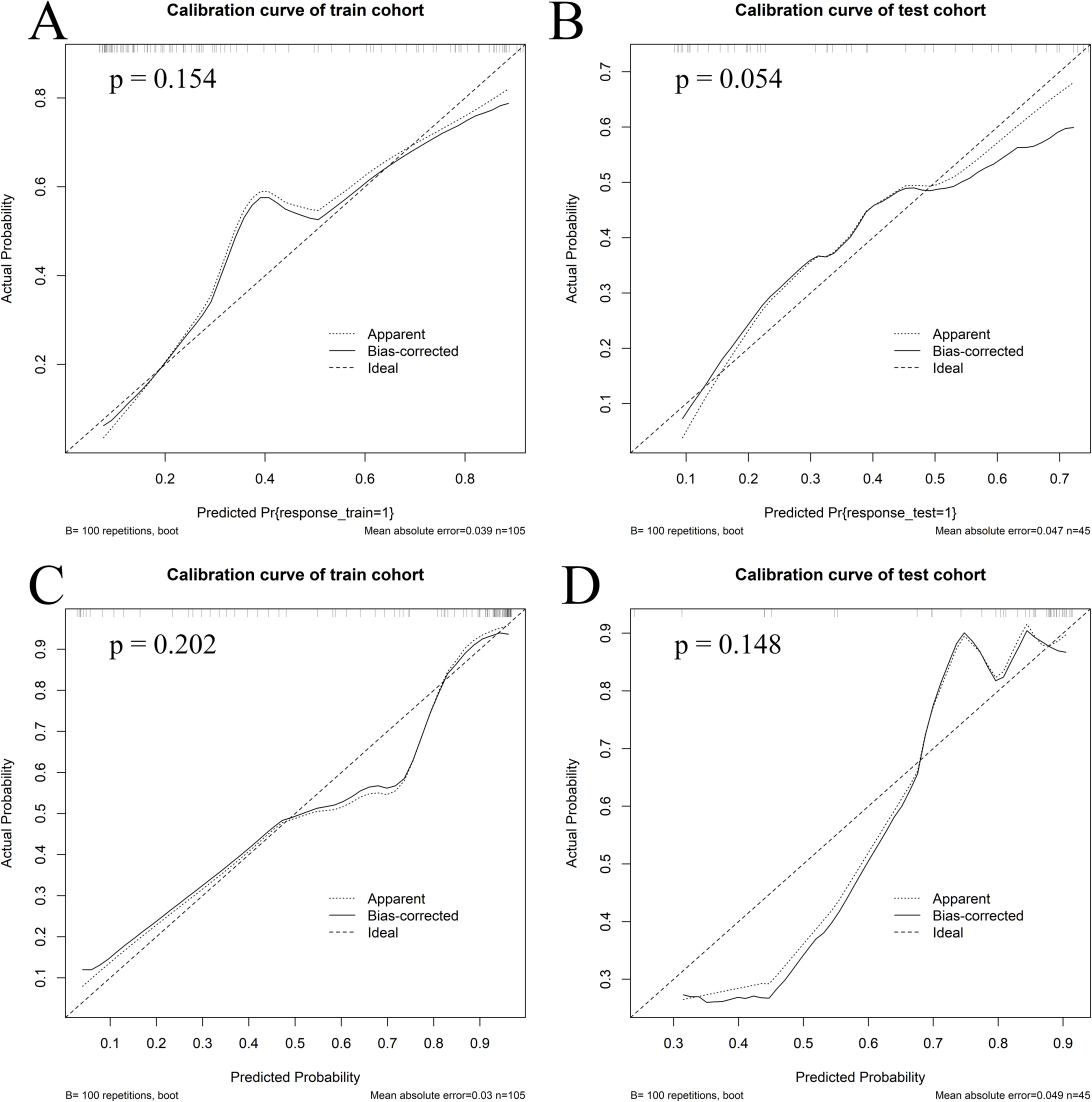
Calibration curves of the combined model. Calibration curves of the combined model in the train **(A)** and test **(B)** cohorts for the first induction chemotherapy cycle, as well as in the train **(C)** and test **(D)** cohorts for the entire induction chemotherapy cycle.

Calibration curves for the combined model showed good agreement between the predicted and observed probabilities of myelosuppression occurrence in both the train and test cohorts for IC - 1 and IC-n (**Figure 5**). Additionally, the Hosmer-Lemeshow test indicated no significant deviation (For IC - 1, P = 0.154 in the train cohort and P = 0.054 in the test cohort, respectively; For IC-n, P = 0.202 in the train cohort and P = 0.148 in the test cohort, respectively), confirming good calibration of the combined model. DCA of the combined model indicated greater clinical net benefit in predicting myelosuppression after IC for LANPC patients compared to either the radiomics or clinics model alone (**Supplementary Figure S1**). These findings suggest that the combined model offers higher clinical utility for predicting myelosuppression compared to the clinics model alone.

## Discussion

In this study, we have developed and internally validated models predicting myelosuppression at different IC stages based on baseline vertebral CaSupp radiomics features combined with clinical characteristics of LANPC patients. As we understand it, this is a pioneering study focused on the prediction of myelosuppression in LANPC, using the CaSupp radiomics derived from DLCT images of bones. Overall, both the radiomics and combined models achieved good predictive performance, with the combined model outperforming the clinical model.

Consistent with previous reports, our observations also demonstrated that patients with relatively abnormal baseline hematological parameters (such as lower Leukocyte, Neutrophil, and Lymphocyte count) (30, 31), lower PNI (32) and older age (13) are more prone to myelosuppression after chemotherapy. Lower baseline Leukocyte and Neutrophil counts reflect insufficient hematopoietic capacity and limited hematopoietic reserves, which make these patients more susceptible to myelosuppression. Chemotherapy agents can impair hematopoietic stem cells or disrupt the hematopoietic microenvironment, leading to a decrease in the ability of bone marrow to generate hematopoietic cells, which in turn causes a rapid decrease in blood cells. Given the short survival time of granulocytes, patients with lower baseline levels of Leukocyte and Neutrophils are more likely to experience myelosuppression or leukopenia after chemotherapy. Older patients are more likely to experience chemotherapy-induced myelosuppression, which may be related to the decline in physical function and reduced hematopoietic capacity in aging individuals. Additionally, elderly patients have lower tolerance to chemotherapy drugs, making them more likely to experience chemotherapy-induced toxicities, such as liver and kidney dysfunction and gastrointestinal reactions. These factors may also affect the metabolism of chemotherapy drugs, leading to drug accumulation in the body, which in turn triggers myelosuppression. PNI reflects the nutritional and inflammatory status of the body. A low PNI value generally reflects poor nutritional status and reduced immune function, which may affect the nutritional support required for hematopoiesis or alter the bone marrow microenvironment. Reportedly, breast cancer patients with low PNI are more likely to develop myelosuppression after neoadjuvant chemotherapy (32), which is consistent with our findings. Nevertheless, although baseline clinical factors can contribute to predicting myelosuppression in this study, their predictive performance remains relatively low (AUC values of 0.687 and 0.652 in the test cohort), suggesting the need to explore better predictive indicators.

Chemotherapy-induced myelosuppression depends on bone marrow reserve function, which is directly related to the condition of bone marrow in cancellous bone, particularly in axial bones such as the lumbar spine and pelvis. Abdominal CT is a routine imaging examination required for clinical staging before treatment in LANPC, making it possible to use lumbar spine CT images to characterize bone marrow status. Unlike conventional CT, DLCT can remove the calcium-induced attenuation from each voxel through material decomposition, allowing simultaneous assessment of trabeculae and marrow in a single scan (33, 34), thus overcoming the limitations of conventional CT in bone marrow imaging. Reportedly, CaSupp imaging can simultaneously evaluate high-contrast (trabecular bone) and low-contrast tissue (bone marrow) in one examination (17, 34). Therefore, CaSupp images hold the potential for qualitative and quantitative diagnosis of bone marrow diseases (35, 36). In DLCT, CaSupp-I can be manually adjusted between 25% and 100% as needed (35). Theoretically, a low CaSupp-I value indicates a high degree of calcium suppression, therefore a low contribution of calcium to the VNCa image. When CaSupp-I is set to 25%, the amount of calcium subtracted reaches its maximum (37). Based on our experience (24), at this CaSupp-I the contribution of calcified components to the vertebral CT images can be completely removed, which means that only the non-calcified components are displayed, primarily including red and/or yellow bone marrow. Therefore, we hypothesized that this CaSupp-I setting may accurately reflect the bone marrow conditions, which was the primary rationale for our selection of this index. Nevertheless, despite a comprehensive review of the current literature, no published studies have, to our knowledge, applied radiomics models based on CaSupp images to predict chemotherapy-induced myelosuppression in NPC patients. As such, whether CaSupp-I of 25% is the optimal choice for radiomics analysis, which is needed in future research.

Radiomics extracts high-throughput features from medical images, hypothesized to reflect patients’ pathophysiological information (14, 38), which provides potential opportunities for using radiomic features to predict toxicities related to anti-tumor therapy. For example, Huang et al. (15) demonstrated that radiomics features extracted from pretreatment cranial and cervical CT images of NPC patients correlated with post-radiotherapy lymphopenia, and the constructed radiomics model achieved high accuracy in predicting grade 4 lymphopenia (ACC = 0.81). Similarly, a radiomics model based on preprocessed pelvic and sacral CT images of cervical cancer can help predict anemia and leucopenia after radiotherapy (16). A previous report found that the variations in texture feature derived from CaSupp images were associated with changes in serum M-protein in myeloma patients (22), suggesting the potential of CaSupp-based radiomics analysis for evaluating hematopoietic status. Our findings preliminarily confirmed the predictive performance of baseline CaSupp-based radiomic features for chemotherapy-induced myelosuppression, with AUC values (0.752 for IC - 1 and 0.740 for IC-n in the test cohorts, respectively) slighter higher than the clinical models. Although the difference in predictive performance between the radiomic and clinical models did not reach statistical significance, the observed trend warrants further investigation. In this study, the combined model outperformed the clinics model, indicating that incorporating radiomic imaging information on top of clinical factors can significantly enhance predictive performance. This is particularly true for IC-n, as the combined model outperformed both the clinics model and the radiomics model. Therefore, in clinical practice, we should make full use of all available information whenever possible. Although mRMR and LASSO were used for feature selection, the performance difference between the train and test cohort was still observed. For both IC - 1 and IC-n, the AUC of the combined model in the test cohort (IC - 1: 0.790; IC-n: 0.806), was notably lower than those in the train cohort (IC - 1: 0.859; IC-n: 0.889), suggesting the possibility of overfitting, and the necessary to increase the sample size and conduct external multi-center validation.

Regarding the four radiomics features (Features 1 to 4) that were most strongly associated with myelosuppression, they may reflect differences in vertebral heterogeneity and bone marrow composition between the myelosuppression and non-myelosuppression groups. Feature 2 represents the mean gray-value of the region (39), and the lower the value, the weaker the signal strength. In this study, a lower value of Feature 2 corresponds to lower CT attenuation in vertebral CaSupp image and therefore a higher proportion of yellow marrow (fatty components) and a lower proportion of red marrow. Features 1 and 4 measure normalized size zone non-uniformity (39), reflecting the variability in region volumes within the ROI. Higher feature value indicates increased heterogeneity in region sizes, implying worse uniformity in texture (39). Feature 3 captures the degree of local variation in image texture, and a lower value is associated with higher tissue heterogeneity (40, 41). In this study, the patients who developed myelosuppression exhibited higher value of Features 1 and 4, as well as lower values of Features 2 and 3, indicating that their vertebrae exhibited greater heterogeneity and higher fat content compared to those without myelosuppression. A more homogeneous microenvironment of bone marrow can enhance the survival and self-renewal capacity of hematopoietic stem cells by reducing intercellular competition and interference (42), and therefore be more beneficial for hematopoiesis.

Our opinion in the differences in vertebral heterogeneity and bone marrow composition between the myelosuppression and non-myelosuppression groups could be supported by the finding in age between the two groups, namely the myelosuppression cohort were older in this study. As age increases, bone marrow heterogeneity also grows (43). This is primarily due to the degeneration and remodeling of the bone marrow microenvironment with aging, characterized by a decrease in osteoblasts and an increase in adipocytes (44). These changes affect the structure and function of bone marrow, and thereby negatively impact the maintenance of hematopoiesis (45). Furthermore, during aging, bone marrow mesenchymal stem cells exhibit altered differentiation abilities, shifting from osteogenesis toward adipogenesis (46), which further leads to increased bone marrow heterogeneity and an increase in fat content.

This is the first study to demonstrate the predictive value of radiomic features extracted from DLCT CaSupp images for predicting chemotherapy-induced myelosuppression in NPC patients. CT was selected as the imaging modality primarily due to its widespread availability and routine application in the metastatic evaluation of NPC. We believe that prediction models based on DLCT CaSupp images may offer a more cost-effective and clinically practical alternative to functional imaging modalities such as positron emission tomography (PET). A previous study reported that dose–volume histogram parameters of red bone marrow derived from MRI images could predict grade ≥2 hematologic toxicity (47). In addition, Dieckmeyer et al. (43) demonstrated the utility of texture analysis for assessing bone marrow heterogeneity. Nevertheless, further research is needed to directly compare the performance of different imaging modalities for bone marrow evaluation.

Of note, the present exploration might have some advantages. First, the CaSupp images were based on DLCT. Considering that DLCT allows retrospective analysis of dual-energy data without requiring a prospective scanning protocol for dual-energy data collection, it is more convenient than other dual-energy CT approaches in clinical practice. Second, the representative trabecular bone region between the midvertebral and the superior endplate of the lumbar vertebra was chosen for its advantages: (1) the lumbar vertebrae are the main site of hematopoiesis in adults, second only to the pelvis, and can reflect the hematopoietic and reserve capacity of the patient’s bone marrow well; (2) this region avoids the higher-density areas in the center of the vertebral body, minimizing the influence of non-bone marrow regions on the results. Finally, we have developed a visual scoring system to quantify the risk of myelosuppression, which may enable personalized prediction for NPC patients undergoing chemotherapy in the future.

However, this study has several limitations. First, the retrospective nature of the study may lead to selection bias. Second, this study was conducted at a single center with a relatively small sample size. The enrolled population may not represent the entire NPC patient population and may not even reflect the demographic characteristics of NPC patients in China. Thus, future multi-center and prospective studies are warranted to construct models with broader generalizability and to confirm their clinical effectiveness. Third, this study solely focused on lumbar spine CT images and did not include data from other axial bones (such as the thoracic spine and pelvis) that may contain information on bone marrow reserve function. The extracted bone marrow information might not fully represent the heterogeneity of bone marrow reserves across different anatomical sites. Therefore, further research is needed to analyze and compare different imaging sites and modalities (such as MRI and PET) to determine the optimal imaging approach. Finally, considering the model’s interpretability and the relatively small sample size, logistic regression was selected as the primary analytical method. Nonetheless, it is necessary to explore more complex machine learning approaches for model construction in future studies.

## Conclusions

In summary, our study developed and validated a radiomics model based on CaSupp images of DLCT, which has the potential to predict the risk of myelosuppression in LANPC patients after chemotherapy. By integrating clinical variables and radiomics features, our model achieved encouraging predictive performance in identifying patients at high risk for myelosuppression. These findings not only confirm the potential of DLCT technology in bone marrow imaging but also provide a potentially valuable tool for clinical use in predicting and possibly preventing chemotherapy-induced myelosuppression.

## Data Availability

The original contributions presented in the study are included in the article/**Supplementary Material**. Further inquiries can be directed to the corresponding author.

## Supplementary material

Supplementary Figure 1 Decision Curve Analysis (DCA) of the models. DCA results of the clinics, radiomics and combined models in the train **(A)** and test **(B)** cohorts for the first induction chemotherapy cycle, as well as in the train **(C)** and test **(D)** cohorts for the entire induction chemotherapy cycle.
