# Supplementary figures and images for: Radiomics model based on vertebral calcium-suppressed CT images for predicting chemotherapy-induced myelosuppression in nasopharyngeal carcinoma
Source: Front Oncol. 2025 Sep 3;15:1574250. doi: 10.3389/fonc.2025.1574250 (PMC12442038; doi:10.3389/fonc.2025.1574250)

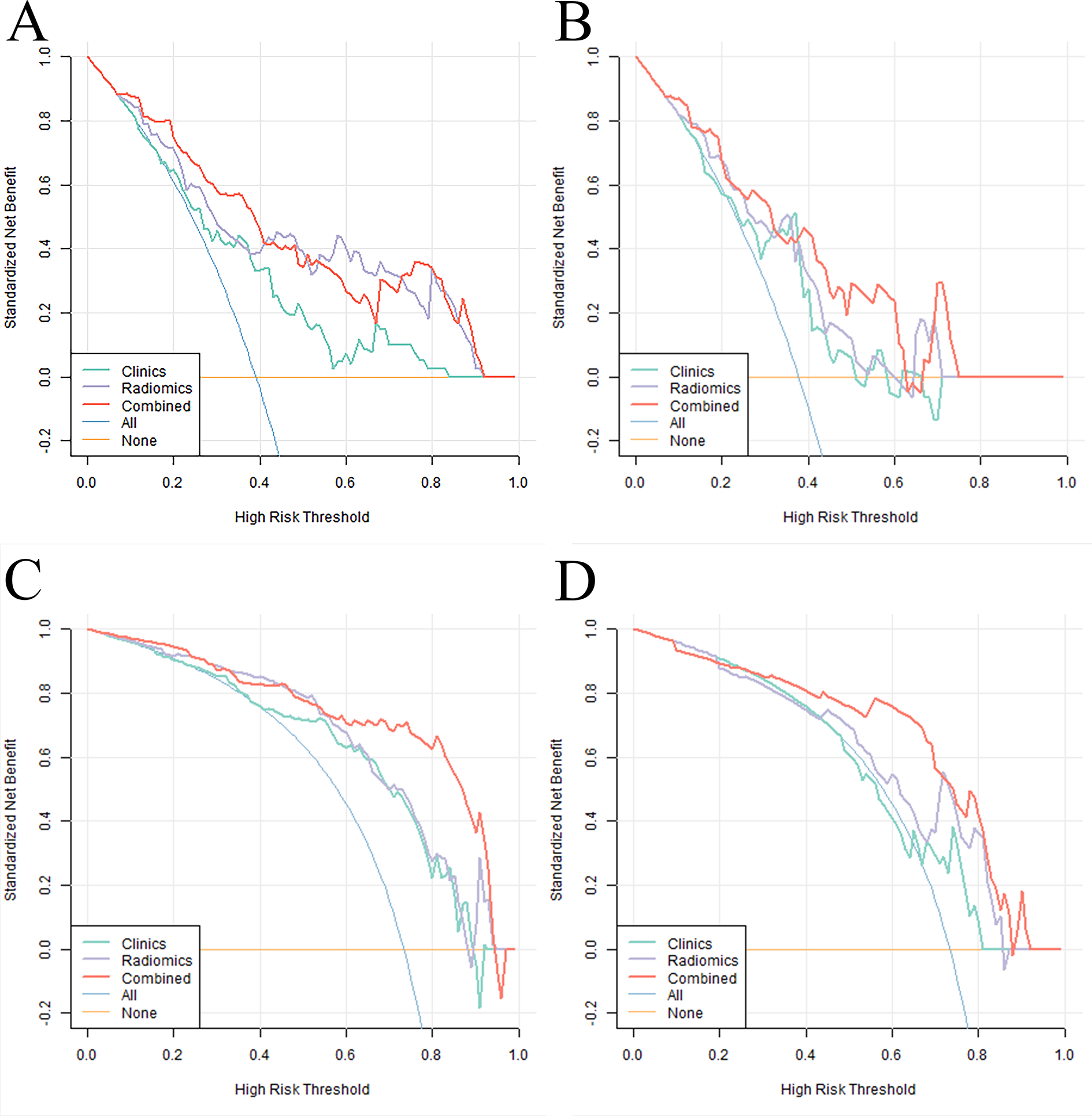

Supplement: Supplementary Figure 1 — Decision Curve Analysis (DCA) of the models. DCA results of the clinics, radiomics and combined models in the train (A) and test (B) cohorts for the first induction chemotherapy cycle, as well as in the train (C) and test (D) cohorts for the entire induction chemotherapy cycle. [file Image1.tif]
