# Supplementary material for: Radiomics model based on vertebral calcium-suppressed CT images for predicting chemotherapy-induced myelosuppression in nasopharyngeal carcinoma
Source: Front Oncol. 2025 Sep 3;15:1574250. doi: 10.3389/fonc.2025.1574250 (PMC12442038; doi:10.3389/fonc.2025.1574250)
Supplement: Supplementary file 2 [file Table1.docx]

Supplementary Table 1. The formula for calculating Rad_score

| Formula | Radiomics features |  | Coefficients |
| --- | --- | --- | --- |
| RS-1= | -0.671 |  |  |
| + | log.sigma.3.0.mm.3D_firstorder_Median | * | -0.395 |
| + | Wavelet.HHL_firstorder_Mean | * | **-0.741** |
| + | original_firstorder_Energy | * | -0.634 |
| + | Wavelet.LLH_gldm_SmallDependenceLowGrayLevelEmphasis | * | -0.378 |
| + | Wavelet.LHL_glcm_Imc2 | * | -0.249 |
| + | Wavelet.HHL_glcm_Idmn | * | -0.161 |
| + | Wavelet.HLL_glszm_LargeAreaHighGrayLevelEmphasis | * | 0.400 |
| + | Wavelet.HHL_glszm_SmallAreaEmphasis | * | 0.314 |
| + | Wavelet.LLH_glszm_SizeZoneNonUniformityNormalized | * | **-0.850** |
| + | log.sigma.5.0.mm.3D_glszm_LargeAreaHighGrayLevelEmphasis | * | 0.293 |
| RS-n= | 1.365737582 |  |  |
| + | Wavelet.LHL_firstorder_Mean | * | 0.385 |
| + | Wavelet.HLL_gldm_SmallDependenceLowGrayLevelEmphasis | * | -0.535 |
| + | Wavelet.LLH_gldm_SmallDependenceLowGrayLevelEmphasis | * | -0.274 |
| + | log.sigma.5.0.mm.3D_gldm_SmallDependenceLowGrayLevelEmphasis | * | -0.043 |
| + | Wavelet.LLH_glcm_Imc1 | * | 0.294 |
| + | Wavelet.LLL_glcm_MCC | * | 0.280 |
| + | original_glcm_InverseVariance | * | **-0.957** |
| + | Wavelet.HLH_glcm_InverseVariance | * | 0.534 |
| + | Wavelet.LHH_glszm_SizeZoneNonUniformityNormalized | * | **-0.700** |
| + | Wavelet.HHL_glszm_ZoneEntropy | * | -0.590 |

RS-1: Formula for calculating first time induction chemotherapy cycle Rad_score; RS-n: Formula for calculating the entire time induction chemotherapy cycle Rad_score.

The bold values represent the top two radiomics features most closely correlated with myelosuppression in IC-1 and IC-n.
