# Supplementary material for: Radiomics model based on vertebral calcium-suppressed CT images for predicting chemotherapy-induced myelosuppression in nasopharyngeal carcinoma
Source: Front Oncol. 2025 Sep 3;15:1574250. doi: 10.3389/fonc.2025.1574250 (PMC12442038; doi:10.3389/fonc.2025.1574250)
Supplement: Supplementary file 3 [file Table2.docx]

Supplementary Table 2. The results of DeLong's test between models

| Diverse comparison | cohort | AUC (95%CI) | P |
| --- | --- | --- | --- |
| First cycle (IC-1) |  |  |  |
| Combined *vs* Clinics | train | 0.859(0.790-0.928) *vs* 0.716(0.614-0.818) | 0.003 |
|  | test | 0.790(0.657-0.922) *vs* 0.687(0.520-0.854) | 0.005 |
| Combined *vs* Radiomics | train | 0.859(0.790-0.928) *vs* 0.825(0.745-0.906) | 0.130 |
|  | test | 0.790(0.657-0.922) *vs* 0.752(0.606-0.899) | 0.163 |
| Radiomics *vs* Clinics | train | 0.825(0.745-0.906) *vs* 0.716(0.614-0.818) | 0.086 |
|  | test | 0.752(0.606-0.899) *vs* 0.687(0.520-0.854) | 0.093 |
| Entire cycle (IC-n) |  |  |  |
| Combined *vs* Clinics | train | 0.889(0.808-0.971) *vs* 0.771(0.658-0.884) | 0.022 |
|  | test | 0.806(0.638-0.973) *vs* 0.652(0.469-0.834) | 0.002 |
| Combined *vs* Radiomics | train | 0.889(0.808-0.971) *vs* 0.824(0.730-0.918) | 0.045 |
|  | test | 0.806(0.638-0.973) *vs* 0.740(0.558-0.922) | 0.027 |
| Radiomics *vs* Clinics | train | 0.824(0.730-0.918) *vs* 0.771(0.658-0.884) | 0.472 |
|  | test | 0.740(0.558-0.922) *vs* 0.652(0.469-0.834) | 0.331 |

95% CI: 95% confidence interval.
